# The role of the *PIK3CA* gene in the development and aging of the brain

**DOI:** 10.1038/s41598-020-79416-6

**Published:** 2021-01-11

**Authors:** Shaozhen Xie, Jing Ni, Hanbing Guo, Victor Luu, Yanzhi Wang, Jean J. Zhao, Thomas M. Roberts

**Affiliations:** 1 Department of Cancer Biology, Dana-Farber Cancer Institute, Boston, MA 02215 USA; 2 Department of Biological Chemistry and Molecular Pharmacology, Harvard Medical School, Boston, MA 02115 USA

**Keywords:** Ageing, Cell signalling

## Abstract

The CLOVES syndrome is an overgrowth disease arising from mosaic activating somatic mutations in the *PIK3CA* gene. These mutations occur during fetal development producing malformation and overgrowth of a variety of tissues. It has recently been shown that treatment with low doses of a selective inhibitor of Class I PI3K catalytic subunit p110α, the protein product of the *PIK3CA* gene, can yield dramatic therapeutic benefits for patients with CLOVES and PROS (a spectrum of PIK3CA-related overgrowth syndromes). To assess the long-term effects of moderate loses of p110α activity, we followed development and growth of mice with heterozygous loss of p110α (*Pik3ca*^+/−^) over their entire lifetimes, paying particular attention to effects on the brain. While homozygous deletion of the *Pik3ca* gene is known to result in early embryonic lethality, these *Pik3ca*^+/−^ mice displayed a longer lifespan compared to their wild-type littermates. These mice appeared normal, exhibited no obvious behavioral abnormalities, and no body weight changes. However, their brains showed a significant reduction in size and weight. Notably, mice featuring deletion of one allele of *Pik3ca* only in the brain also showed gradually reduced brain size and weight. Mechanistically, either deletion of p110α or pharmacological inhibition of p110α activity reduced neurosphere size, but not numbers, in *vitro*, suggesting that p110α activity is critical for neuronal stem cells. The phenotypes observed in our two genetically engineered mouse models suggest that the sustained pharmacological inhibition of the PIK3CA activity in human patients might have both beneficial and harmful effects, and future treatments may need to be deployed in a way to avoid or minimize adverse effects.

## Introduction

CLOVES (congenital lipomatous overgrowth, vascular malformations, epidermal nevi, scoliosis/skeletal and spinal anomalies) syndrome is a rare overgrowth disorder^1,2^. CLOVES syndrome belongs to a group of overgrowth syndromes that are collectively referred to as *PIK3CA*-related overgrowth spectrum (PROS) that are caused by somatic activating mutations in the gene encoding the phosphatidylinositol 3-kinase (PI3K) catalytic subunit alpha (*PIK3CA*) occurring early during embryogenesis^3,4^. The class IA PI3Ks consist of a p110 catalytic subunit and a p85 subunit. The two ubiquitously expressed p110 catalytic isoforms, p110α and p110β, are encoded by *PIK3CA* and *PIK3CB* respectively. PI3K activity is important for cellular responses to growth factors, including the fibroblast growth factor and insulin-like growth factor pathways. The small family of class IA PI3Ks convert phosphatidylinositol (4,5)-bisphosphate (PIP2) to phosphatidylinositol (3,4,5)-trisphosphate (PIP3), a potent second messenger that can activate multiple effector proteins, including AKT (also known as protein kinase B, PKB), to regulate many biological processes such as cell proliferation, cell growth, survival, and metabolism^5–7^.

The insulin/IGF-1 (insulin-like growth factor 1)/PI3K/AKT/mTOR(mammalian target of rapamycin) /FOXO (forkhead box O) signaling pathway is one of the most important pathways influencing longevity and aging, acting through many of the downstream effectors to control a variety of biological functions, including endocrine regulation, energy expenditure, metabolism, stress resistance, response to environmental cues, and both male and female reproduction^8–11^. PI3K signaling is essential for growth and food storage. In the nematode C. elegans, mutations inactivating age-1, the gene encoding the single Calss 1A catalytic subunit of PI3K, result in a significant extension of lifespan^12,13^ . In mice total body knockout of either the insulin receptor or the ubiquitously expressed class IA PI3K isoforms results in early lethality in mice^14,15^. Yet partially inhibiting activities of many of the PI3K pathway components paradoxically lengthens life span. For example, mutations that decrease IGF-1 or growth hormone signaling limit body size and prolong lifespan in mice^16^, and mice with hypomorphic PI3K activity also showed reduced body weight and increased longevity^17^. Partial inactivation of insulin-like growth factor 1 receptor (IGF-1R) in the embryonic brain caused growth retardation, smaller adult size, and longer mean lifespan^18^. The site of pathway downregulation can also matter. Thus, while mice lacking the insulin receptor in liver develop diabetes and die early, mice lacking the insulin receptor in adipose tissue live longer^19^.

Here we generated two mouse models featuring heterozygous loss of p110α expression and found that the lifespans of mice with whole body loss of one copy of *Pik3ca* had significantly longer lifespans than littermate controls. Surprisingly, both *Pik3ca*^+/–^ mice and mice lacking one allele of *Pik3ca* in the brain alone have smaller brains, suggesting that sustained pharmacological inhibition of the PIK3CA activity in human patients might have both beneficial and harmful effects.

## Results

### Male Pik3ca^+/−^ mice live longer

**Figure 1:**
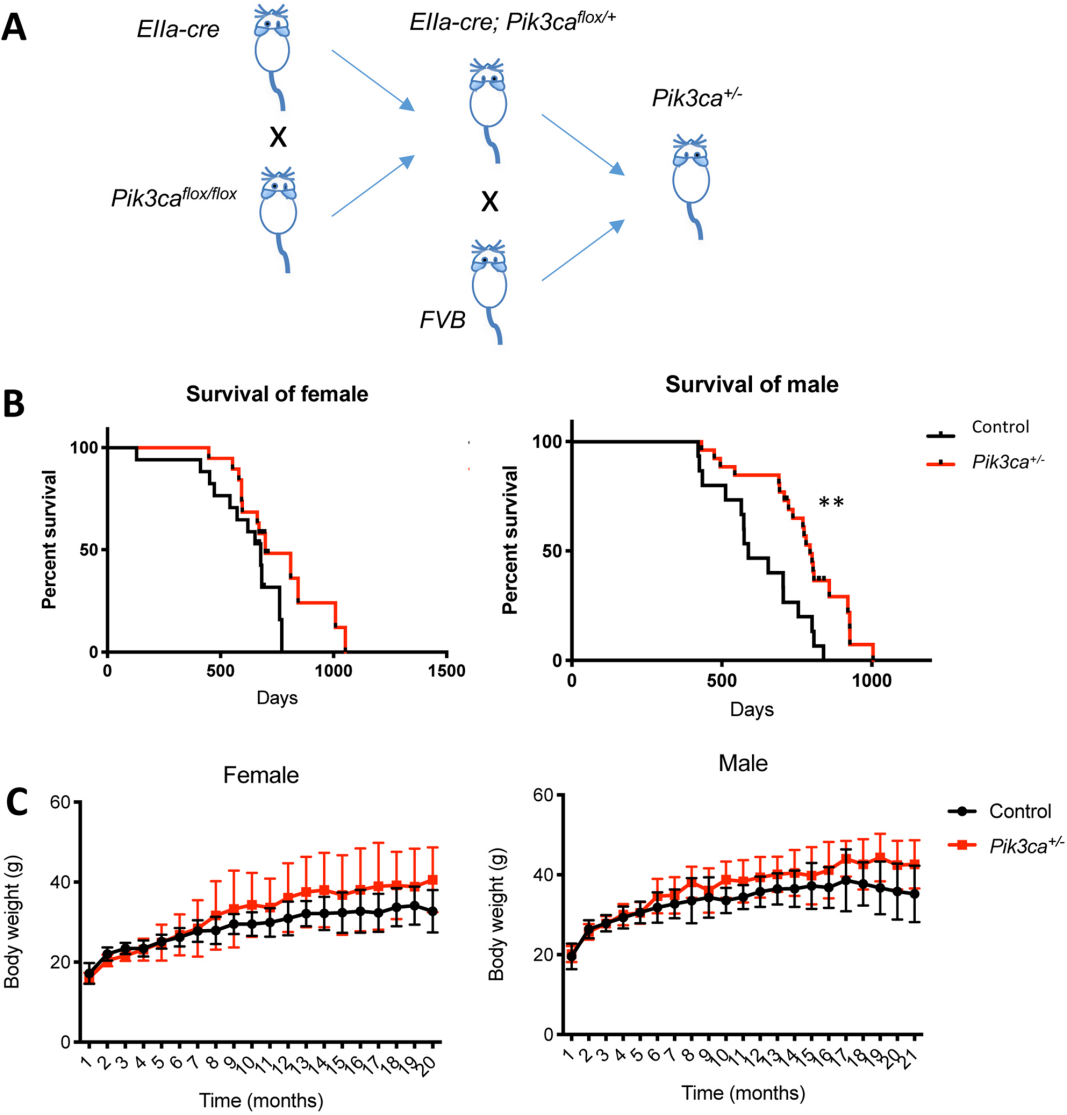
*Pik3ca*^+*/−*^ mice live longer. (**A**) Schematic flow chart for generating *Pik3ca*^+*/−*^ mice. (**B**) Kaplan–Meier survival curves of *Pik3ca*^+*/−*^ mice (red line) and control mice (black line) (left panel: female; right panel: male). **P < 0.01 (**C**) Body weight of mice at different ages. Mice of both genders (left panel: female; right panel: male) were monitored monthly. Data represents mean ± SD ( n = 5–16 at each time point).

Mice featuring whole body homozygous deletion of *Pik3ca* die early in embryonic development^14^. To assess long term impact of moderate loses of PI3K activity, we generated mice with heterozygous loss of *Pik3ca* using a Cre-LoxP system. Mice carrying a Cre transgene under the control of the adenovirus *EIIa* promoter, which targets expression of Cre recombinase to most tissues including germ cells, were mated with *Pik3ca*^*flox/flox*^ mice^20^ to generate *EIIa-cre; Pik3ca*^*flox/*+^*mice*. To avoid the potential side effects of Cre expression in mice, we further back crossed these mice with wild type FVB mice to remove the *EIIa-cre* transgene (Fig. 1A). The resulting mice that are heterozygous for p110α were then monitored during their entire life span, together with their wild type littermates. Notably, male mice heterozygous for p110α showed a significant increase in longevity with median survival prolonging roughly 35% from ~ 588 to ~ 794 days (Fig. 1B). While female heterozygous mice also showed strong tendency to prolonged survival, it was not statistically significant at this time with a small sample population. A sex specific increase in lifespan has been seen in many mouse models for aging and longevity^17,21,22^. Surprisingly, unlike the hypomorphic PI3K mice^17^ and many other long-lived mutant mice, neither male nor female *Pik3ca*^+*/−*^ mice had decreased body weights compared to their wild type littermates (Fig. 1C). Our data thus far suggest that loss of one allele of p110α mice may prolong the healthy life span.

### Pik3ca^+/−^ mice have substantially reduced brain weight and size

**Figure 2:**
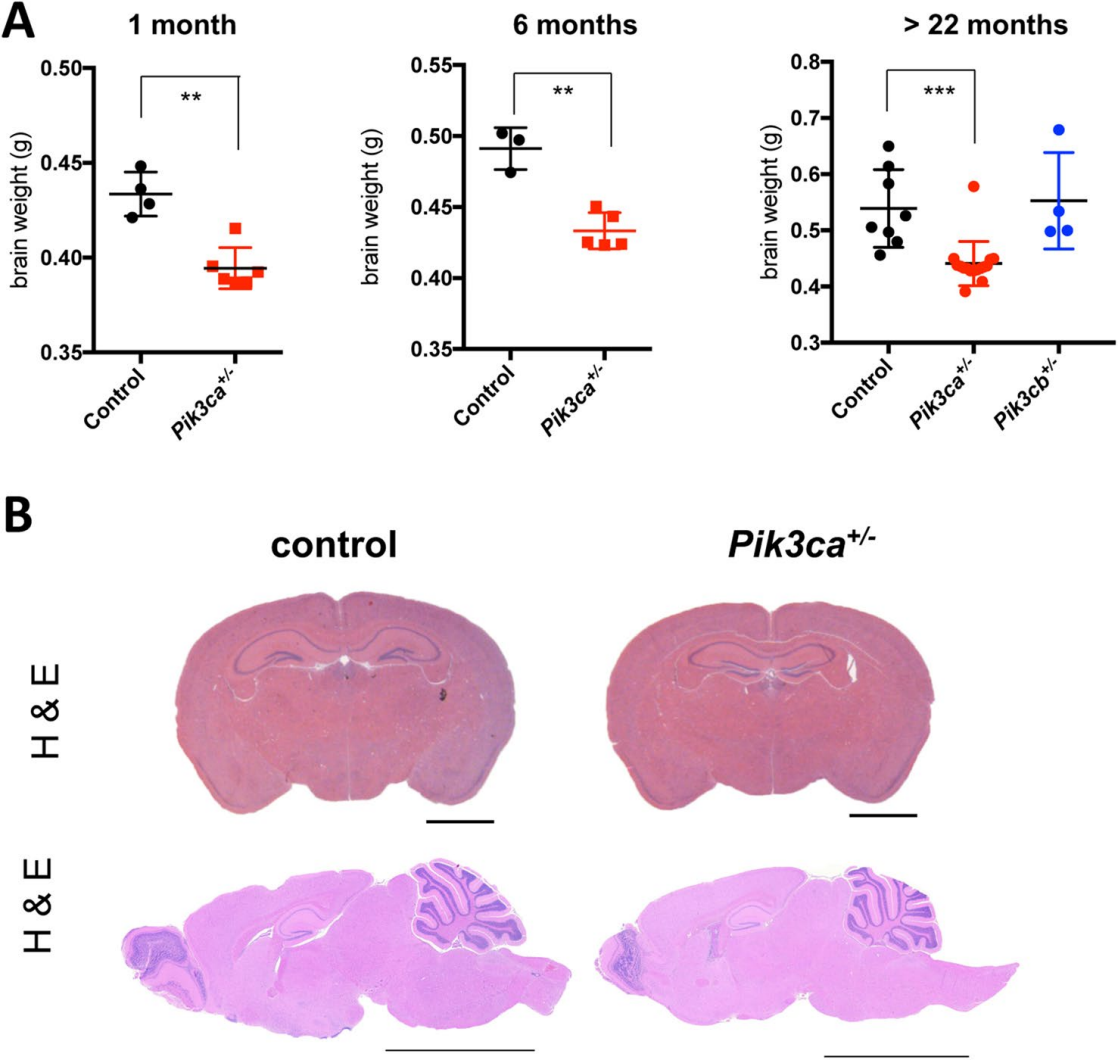
*Pik3ca*^+*/−*^ mice have substantially reduced brain weight and size. (**A**) Brain weight of mice at different ages. Data represents mean ± SD (n = 3–16). **P < 0.01, ***P < 0.001 (**B**) H&E staining in the indicated mice. Upper panel (coronal section), > 22 months old. Scale bar, 2 mm. bottom panel (sagittal section), 12 months old, scale bar, 5 mm.

The effects of genes on lifespan may be dependent on their activity in specific tissues, including fat, liver, muscle, and the central nervous system^18,19^. Although *Pik3ca*^+*/−*^ mice displayed normal body weight and appeared normal with respect to the morphologies of most organs, our previous studies on the effects of homozygous deletion of p110α in the brain (see below) led us to determine if their brains were normal as well. Brains were dissected from *Pik3ca*^+*/−*^ mice and wild type controls and weighted at different ages. Surprisingly, brain weights of *Pik3ca*^+*/−*^ mice were about 9% lower than that of wild type littermates at 1 month, about 13% at 6 months, and about 18% at 23 months (Fig. 2A). Notably, histological analysis of the *Pik3ca*^+*/−*^ brains did not reveal any abnormal structures (Fig. 2B). Together, these results indicated that *Pik3ca*^+*/−*^ mice appear normal and live significantly longer with reduced brain weight and size.

### GFAP-cre; Pik3ca^flox/+^ mice have substantially reduced brain weight and size

**Figure 3:**
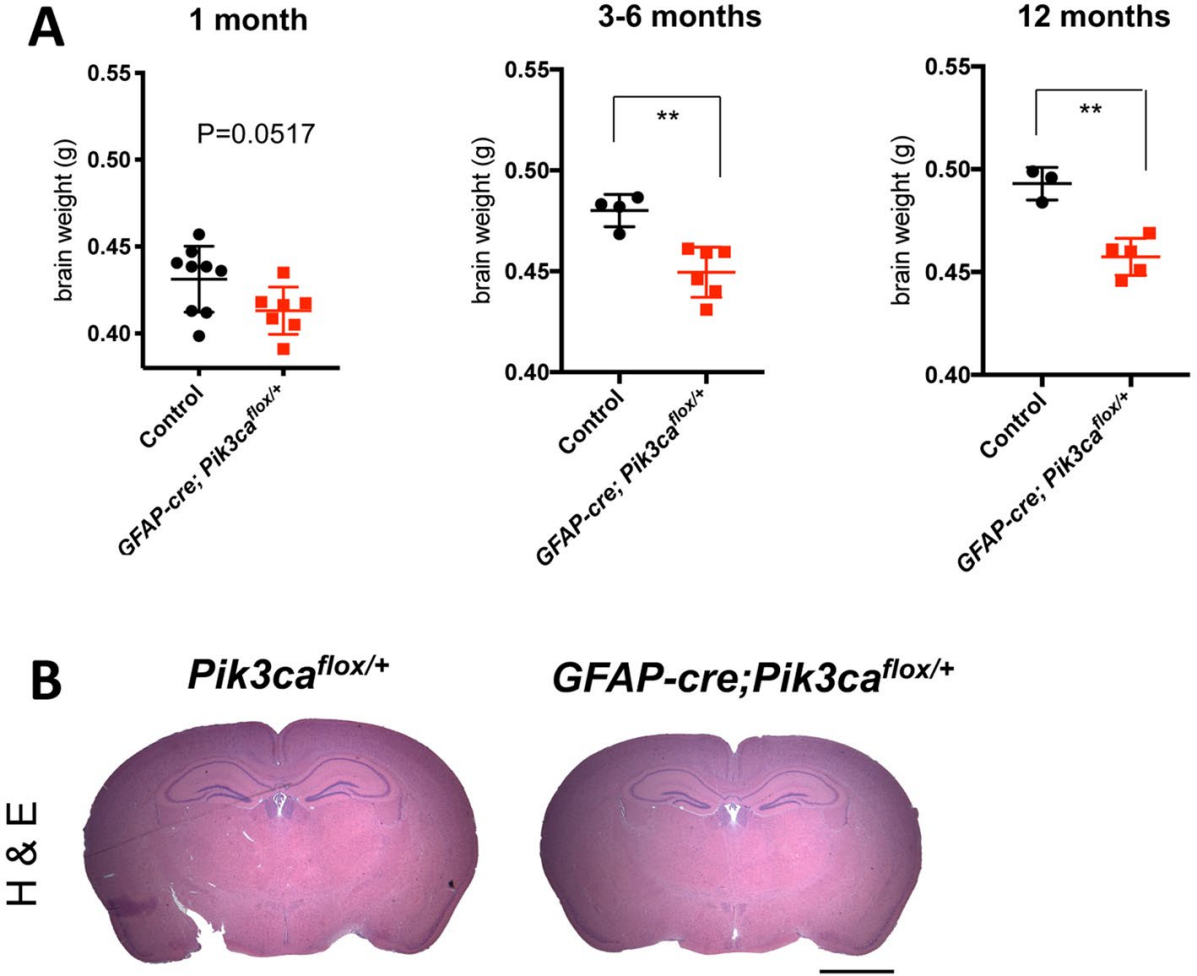
*GFAP-cre; Pik3ca*^*flox/*+^ mice have substantially reduced brain weight and size. (**A**) Brain weight of mice at different ages. Data represents mean ± SD ( n = 3–9). **P < 0.01. (**B**) H&E staining in the indicated mice at 6 months old. Scale bar, 2 mm.

We wondered if the effects seen in the brains of *Pik3ca*^+*/−*^ mice resulted from the organ specific loss of p110α activity or were due, at least in part, to p110α loss elsewhere in the body. We therefore tested whether deleting one allele of *Pik3ca* in the brain alone would be sufficient to affect brain size in mice. We generated mice with brain-specific knock out of one allele of *Pik3ca* by mating transgenice mice with a brain specific glial fibrillary acidic protein (*GFAP)* promoter driving Cre expression (*GFAP-cre*) with *Pik3ca*^*flox/flox*^ mice to generate *GFAP-cre; Pik3ca*^*flox/*+^ mice. As shown in Fig. 3A, mice heterozygous for p110α expression in the brain had smaller brains than the wild type littermates as young adults. The brain weights of *GFAP-cre; Pik3ca*^*flox/*+^ mice were also about 9% reduced as compared to those of wild type littermates at ages 3 months to 12 months. Notably, histological analysis of the brains of these mice did not reveal any abnormal structures (Fig. 3B).

We also generated *GFAP-cre; Pik3ca*^*flox/flox*^ mice in which both alleles of *Pik3ca* were knocked out in the brain. As expected, mice with homozygous deletion of p110α in the brain had significantly smaller brains than the wild type littermates at as early as 2 weeks of age. (Supplemental Fig. S1A). Furthermore, we observed specific structural changes in hippocampus in the *GFAP-cre; Pik3ca*^*flox/flox*^ mice (Supplemental Fig. S1B). Our findings thus suggest there might be detrimental effects on the brain with more complete pharmacological inhibition of p110α.

### Loss of p110α inhibits neural stem/progenitor cells (NSC/NPCs) proliferation

**Figure 4:**
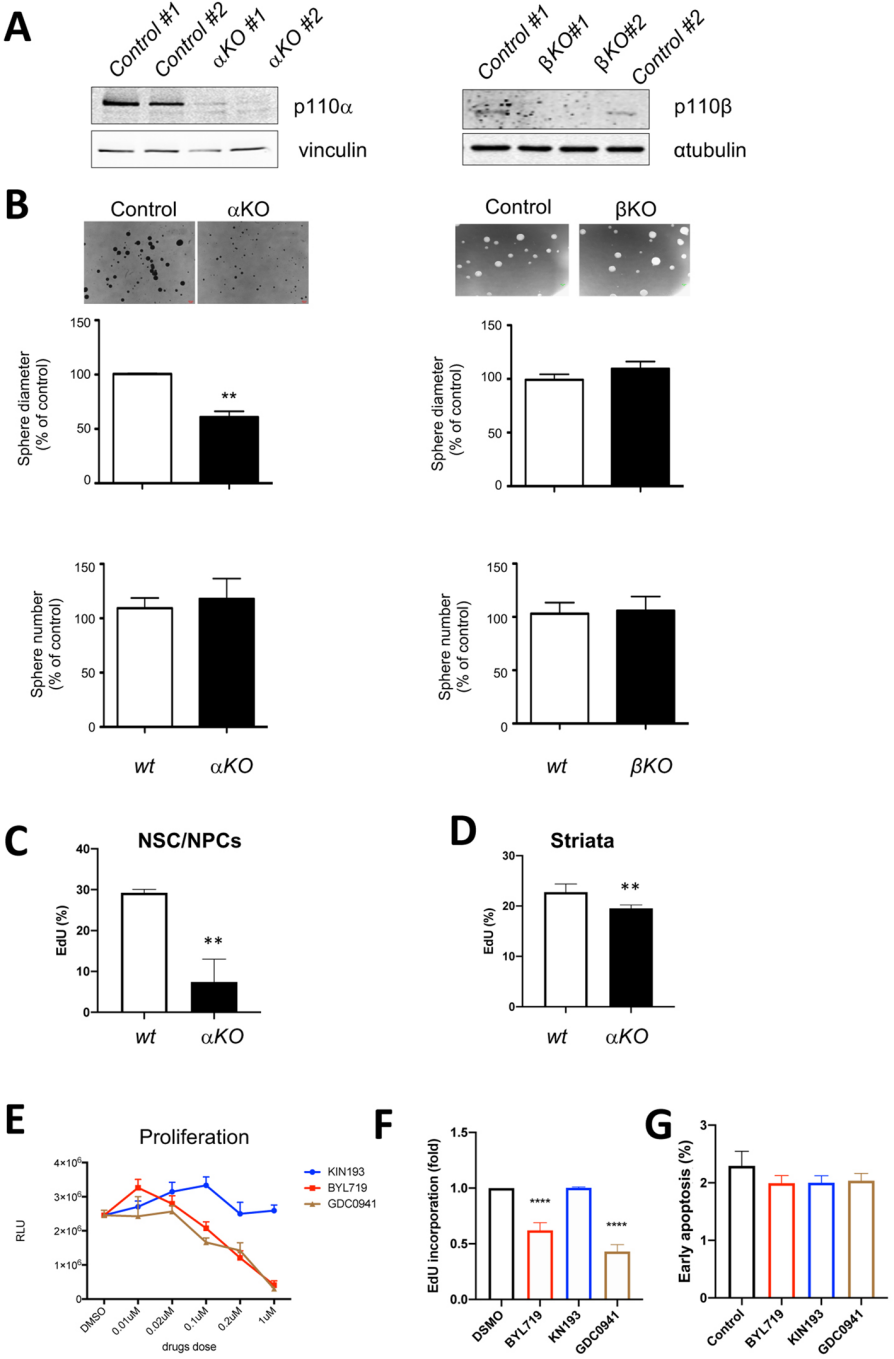
Ablation of p110α, but not p110β, reduces the neurosphere proliferation using primary cultured neural stem/progenitor cells (NSC/NPCs). (**A**) Immunoblotting of p110 expression in embryonic NSC/NPCs isolated from wild type (*Pi3kca*^*f/f*^*, Control*) and αKO (*GFAP*+*; Pi3kca*^*f/f*^) littermates (left panel); or wild type (*Pi3kcb*^*f/f*^*, Control*) and βKO (*GFAP*+*; Pi3kcb*^*f/f*^) littermates (right panel). (**B**) Representative image of neurospheres cultured for 7 days (top panel). Quantification of neurosphere size (middle panel) and neurosphere number (bottom panel). Data represents mean ± SEM (n = 4–6). **P < 0.01. (**C**) EdU incorporation in NSC/NPCs. Cells were labedled with EdU (10 μM) for 60 min. Data represents mean ± SD (n = 3). **P < 0.01. (**D**) EdU incorporation in striata cells. Pregnancy mice were injected with EdU (5 mg/ml, 300 μl) and euthanized 4 h later to collect E14.5 embryonic striata and EdU + cells were determined by flow cytometry. Data represents mean ± SD (n^wt^ = 3, n^αKO^ = 7). **P < 0.01. (**E**) Cell viability of wild type NSC/NPCs treated with BYL719 (a p110α inhibitor), KIN193 (a p110β inhibitor), or GDC0941 (a pan p110 inhibitor) for 3 days. Proliferation were accessed by CelltiterGlo. Data represents mean ± SEM (n = 3). (**F**) EdU incorporation in wild type NSC/NPCs treated with indicated compounds (1 μM) for 3 days. Cells were labelled with EdU (10 μM) for 90 min. Data represents mean ± SEM (n = 6). ****P < 0.0001. (**G**) Early Apoptosis of NSC/NPCs with the indicated compounds (1 μM) treatment for 3 days measured by Annexin V+/PI− staining and flow cytometry. Data represents mean ± SD (n = 3).

Neural stem/progenitor cells (NSC/NPCs) are self-renewing and multi-potent cells of the CNS. The regulation of NSC/NPC’s proliferation and differentiation is key to brain development^23^. The *GFAP-cre* transgenic line in this study utilizes a human GFAP promoter which is active in NSC/NPCs in the brain prior to embryonic day e14.5^24,25^. To determine roles of p110α and p110β in NSC/NPCs proliferation, we performed neurosphere assays on NSC/NPCs isolated from the brain subventricular zone (SVZ) of E14.5 embryos of *GFAP-cre; Pik3ca*^*flox/flox*^(αKO*), GFAP-cre; Pik3cb*^*flox/flox*^ (βKO) and their littermates. As shown in Fig. 4A,B, loss of p110α resulted in ~ 40% decreased NSC/NPC sphere size but had no effect on sphere number. In contrast, loss of p110β did not change NSC/NPC size or number compared to their wild type littermates. Furthermore, the effect of the p110α on cellular proliferation was examined by an EdU incorporation assay. Compared to wild-type controls, p110αKO NSC/NPCs had a significant decrease in EdU incorporation (~ 29% vs ~ 7%) (Fig. 4C). Consistently, the wild-type E14.5 embryonic striata, where NSC/NPCs are enriched in the brain, had more EdU incorporation than the p110αKO embryonic striata had (Fig. 4D). Collectively, these results show that p110α is implicated in brain development via a role in NSC/NPC growth.

We next applied pharmacological approaches to explore specific roles of p110α and p110β in NSC/NPCs by treating wild type NSC/NPCs with isoform-specific or pan-PI3K small molecule inhibitors. Consistent with our genetic models, treatment of wild type NSC/NPCs with BYL719 (alpelisib, a p110α-selective inhibitor), as well as GDC0941 (a pan-PI3K inhibitor), but not KIN193 (a p110β-selective inhibitor), led to a decrease in NSC/NPCs viablity (Fig. 4E). Similarly, the p110α-selective inhibitor and pan-PI3K inhibitor, but not the p110β-selective inhibitor, dramatically blocked NSC/NPCs proliferation as examined by EdU incorporation (Fig. 4F). Interestingly, none of above PI3K selective or pan inhibitors affected NSC/NPCs apoptosis in an annexin V apoptosis assay (Fig. 4G), suggesting that decreased cell proliferation by p110α inhibition might not be due to an increase in cell death. Together, results of both genetic deletion and pharmacological inhibition of p110α suggested that p110α may be critical in brain development via a role in NSC/NPC growth.

## Discussion

The PI3K/AKT/mTOR pathway is one of the most frequently dysregulated pathways in cancer and consequently, numerous compounds that target key components of this signaling pathway have been clinically tested in a range of different cancers. Notably the clinical development of many of these agents, either singly or in combination, has stalled due to prohibitive on target toxicities^26,27^. Recently, alpelisib became the first PI3K p110a inhibitor approved by FDA to treat *PIK3CA*-mutated, advanced or metastatic breast cancer^28^, but dosing of this compound too has been limited by on target toxicities in normal organs. However, alpelisib treatment at relatively low doses rescues the PROS phenotype in a mouse model, and has proved to be remarkably effective with a promising safety profile in a small cohort of patients with PROS^29^. This recent development will inevitably result in more PROS/CLOVES patients receiving p110α inhibitors. Thus it is important to understand long-term effects of p110α inhibition. Common side effects for alpelisib in treating cancer patients include hyperglycemia, kidney problems, diarrhea, rash, low white blood cell counts, liver problems, pancreatitis, vomiting, and hair loss^28^. Therefore, a reduced dose of alpelisib, as compared to that given to cancer patients, is essential for patients with PROS; and indeed the currently used dosages for patients with overgrowth syndromes are much lower than those used for cancer therapy. In this communication, our genetic mouse models provide a platform to help predict the long term effects of partial inhibition of p110α, allowing us to follow development and growth of the whole body in heterozygous p110α (*Pik3ca*^+*/−*^) mice and the brain in brain-specific heterozygous p110α (*hGFAP-cre; Pik3ca*^*flox/*+^) mice over their entire lifetimes. Our results suggest that partial loss of p110α signaling would generally not have any adverse effects over an entire lifetime. Indeed, as has been previously suggested, partial downregulation of this pathway could be beneficial^17,21,22^. However our particular emphasis on role of p110α in the brain has revealed a potentially deleterious effect of even partial p110α loss in the brain.

Early studies suggested that loss-of-function mutations in p110α impair insulin signaling causing insulin resistance and inducing a pre-diabetic state^17,21^. Mice heterozygous for the kinase-dead D933A allele of p110α (p110α^D933A/WT^) mice are hyperphagic and exhibit higher adiposity, insulin resistance and glucose intolerance at a young age. Interestingly, aged p110α^D933A/WT^ mice are protected from age-related reductions in insulin sensitivity, glucose intolerance and fat accumulation, and exhibit a slightly extended lifespan in male mice (by 6% compared to wt littermates^17,21^. Here our male p110α^+/−^ mice also showed an extended lifespan (more than 35% compared to wide-type littermates). Importantly, p110α^D933A/WT^ mice showed reduced body weight that indicated a growth retarding effect of abnormal insulin signaling and metabolism^17,21^. In contrast, body weights of p110α^+/−^ mice were normal throughout their whole life span, as recorded over a period of 21 months. These results suggest that partial inhibition of p110α may have a smaller benefit in terms of lifespan than changing the ratio of p110β and p110α activation upon physiological stimulation of insulin/IGF signaling.

Our results suggest that the beneficial effects of p110α inactivation/loss that result in a longer lifespan are, however, accompanied with potentially harmful effects on brain size. Despite the fact that p110α^+/−^ mice appear normal, reduced brain size could potentially have adverse consequences on cognition and other biological functions. The brain size reduction in p110α^+/−^ mice likely starts during embryonic development. It remains unclear if pharmacologically reducing PI3K signaling will have the same potent effect on the adult brain as we have observed here. Notably, GFAP-cre mediated ablation of one allele of *Pik3ca* in mice did not lead to significantly reduced brain weights at one month. However the brain-specific loss of one allele of *Pik3ca* mice did result in reduced brain weight in young adults, suggesting that chronic pharmacological inhibition of p110α might affect brain functions in human adults, affecting either stem/progenitor cells, differentiated cells, or both.

The beneficial effect of p110α specific inhibition in CLOVES/PROS patients is a triumph of targeted therapies. Our results coupled with previous studies suggest that partial pharmacological inhibition of p110α activity should be relatively benign in terms of long term on-target side-effects. However our emphasis on studying the effects of reducing p110α activity in the brain does suggest an important caveat, in that partial loss of p110α function could result in significant and potentially adverse effects on the brain. Specifically our results suggest that consideration should be given to the ability of p110α targeted therapies to cross the blood brain barrier in CLOVES/PROS patients. The blood–brain barrier (BBB) proper is composed of endothelial cells of the cerebral microvasculature, which are interconnected by tight junctions that in turn form a physical barrier restricting paracellular flux. Tight control of vascular permeability is essential for the homeostasis and functionality of the central nervous system. While some PI3K inhibitors can cross the BBB, alpelisib is not known to be BBB-penetrant. However, alpelisib is likely to cross the BBB when BBB is damaged, which frequently happens in the cancer patients, or when BBB is immature as in new-born babies. Notably, alpelisib has shown activity in PROS patients with brain abnormalities, including hemimegalencephaly^29^. This result is likely due to the reduced efficacy of the BBB in the brains of patients featuring CLOVES/PROS mutations in brain tissues. Our results suggest that the brain specific, potentially deleterious effects of long term, low dose treatment with PI3K inhibitors should be carefully monitored.

## Materials and methods

### Animals

*Pik3ca*^*flox/flox*^ mice (FVB strain) were first crossed with *EIIa-cre* mice (FVB, Jackson lab #003314) to generate *EIIa-cre* + *; Pik3ca*^*flox/*+^. Those mice were then crossed with FVB mice to remove *EIIa-cre* and generate *Pik3ca*^+*/−*^ mice. *Pik3ca*^*flox/flox*^ (FVB) mice were crossed with *hGFAP-cre* (FVB, Jackson lab #004600) mice to generate *hGFAP-cre; Pik3ca*^*flox/*+^ mice. Mice were housed under standard conditions and monitor closely for systemic symptoms of morbidity, including inability to obtain food or water, bloating or other humane endpoints necessitating euthanasia. All animal experiments were performed in accordance with NIH animal use guidelines, and all protocols were approved by the Dana-Farber Cancer Institute Animal Care and Use Committee.

### Primary neural stem and progenitor cell culture

Primary neural stem and progenitor cells (NSCs/NPCs) were microdissected from the brain of E14.5 mice and mechanical digestion as previously described^24,25^. The cells were maintained in NeuroCult Proliferation Kit (Stemcell Technologies) supplemental with 20 ng/ml EGF.

### EdU incorporation assay

For in vitro EdU incorporation assay, NSC/NPCs were treated with EdU (10 μM) for 60 m or 90 m. After labeling, cells were collected and stained with Click-iT EdU Alexa Fluor 647 Flow Cytometry Assay Kit (Life Technologies #C10424) according to the manufacturor’s instruction.

For in vivo EdU incorporation assay, E 14.5 pregnant mice were administered EdU (5 mg/ml, 300 μl) through intraperitoneal injection. 4 h after injection, mice were euthanized and E14.5 embryonic striatas in the brain were isolated and stained with Click-iT EdU Alexa Fluor 647 Flow Cytometry Assay Kit (Life Technologies #C10424) according to the manufacturor’s instruction. EdU amount was determined by flow cytometry on JF Fortessa HTS (BD Biosciences). Data were analyzed using FlowJo software (version 10).

### Annexin V apoptosis assay

Cells were stained with FITC Annexin V (Biolegend #640906) in Annexin V binding buffer (Biolegend #422201) with propidium iodide (PI, final 0.05 mg/ml) for 15 min at room temperature according to manufacturer’s instruction. Early apoptosis (FITC+ PI−) was detected by flow cytometry on JF Fortessa HTS (BD Biosciences). Data were analyzed using FlowJo software (version 10).

### Cell proliferation assay

Cells were seeded in 96-well plates at a density of 1000 cells/well and treated with different compounds for 3 days. Cell viability was assessed by Celltiter-Glo assay (Promega) according to manufacturer’s instruction.

### Compounds and reagents

BYL719, KIN193, and GDC0941 were obtained from MedChemexpress (Shanghai, China). Anti-p110α (#4249) and anti-p110β (#3011) antibodies were obtained from Cell Signaling Technology (Hanover, MA). Anti-α-Tubulin (T9026) and anti-Vinculin (v9131) antibodies were obtained from Sigma-Aldrich (Rockford, IL).

### Western blot analysis

Western blot analysis was performed as described previously^30^.

### Statistical analysis

Unpaired Student’s t-test or ANOVA by GraphPad Prism (GraphPad Software) was performed to determine statistical significance. Data are considered significant when *P* values are < 0.05.

## Supplementary Information

Supplementary Figures.
