# Supplementary material for: The role of the PIK3CA gene in the development and aging of the brain
Source: Sci Rep. 2021 Jan 11;11:291. doi: 10.1038/s41598-020-79416-6 (PMC7801510; doi:10.1038/s41598-020-79416-6)
Supplement: Supplementary file 1 — Supplementary Figures. [file 41598_2020_79416_MOESM1_ESM.pdf]

Supplementary materials

The role of the *PIK3CA* gene in the development and aging of the brain

Shaozhen Xie[1,2,3], Jing Ni[1,2,3], Hanbing Guo[1,2], Victor Luu[1,2], Yanzhi Wang[1,2], Jean J. Zhao[1,2,*], Thomas M. Roberts[1,2,*]

[1]Department of Cancer Biology, Dana-Farber Cancer Institute, Boston, MA 02215, USA.

[2]Department of Biological Chemistry and Molecular Pharmacology, Harvard Medical School, Boston, MA 02115, USA

[3]These authors contributed equally

*Correspondence to: Jean J. Zhao (email: jean_zhao@dfci.harvard.edu); and Thomas M. Roberts, PhD (email: thomas_roberts@dfci.harvard.edu)

SUPPLEMENTARY FIGURES

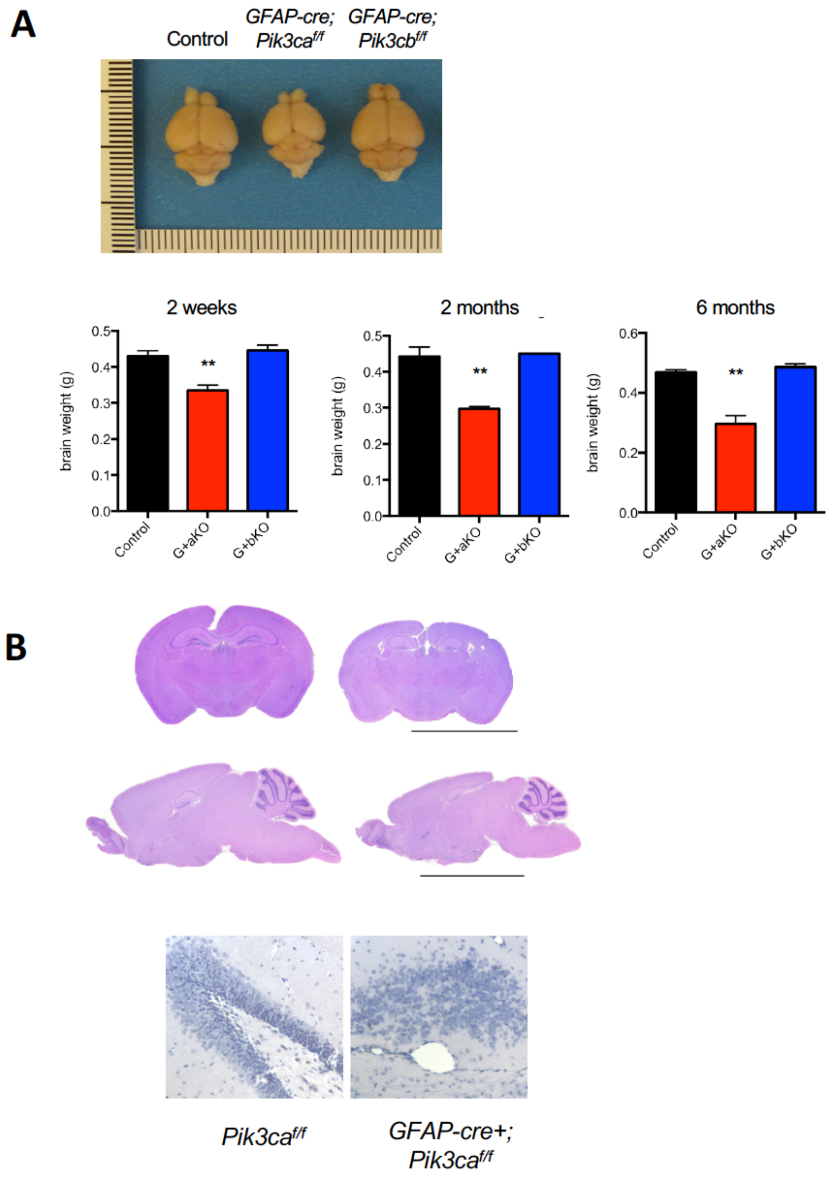

Supplemetary Figure 1. The mice with brain-spcific 110α knockout, but not p110β, have significantly smaller and abnormal brains. (A) Overall brain picture and brain weight of *GFAPcre;Pik3ca*$^{f/f}$ mice (red bar), *GFAPcre;Pik3cb*$^{f/f}$ mice (blue bar), or wild type control mice (black line) at different ages. Bars represents mean ± SEM (n ≥ 5). (B) H&E staining of coronal sections (upper panel, 12 months old), sagittal sections (middle panel, 2 months old), and hippocampus region (lower panel) of the mouse brain tissue. Scale bar, 5 mm

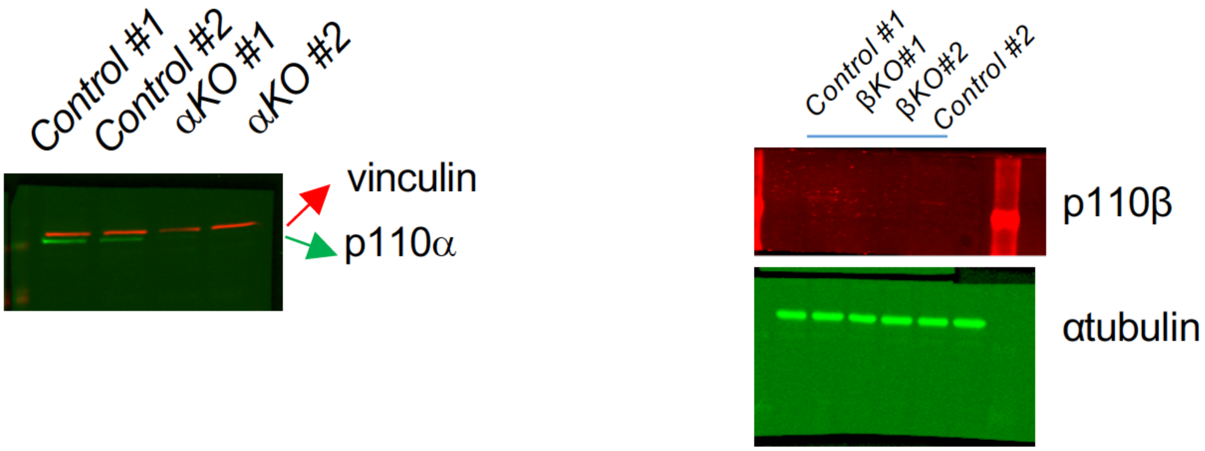

Supplemetary Figure 2. Uncropped images from Western blot related to Figure 4A

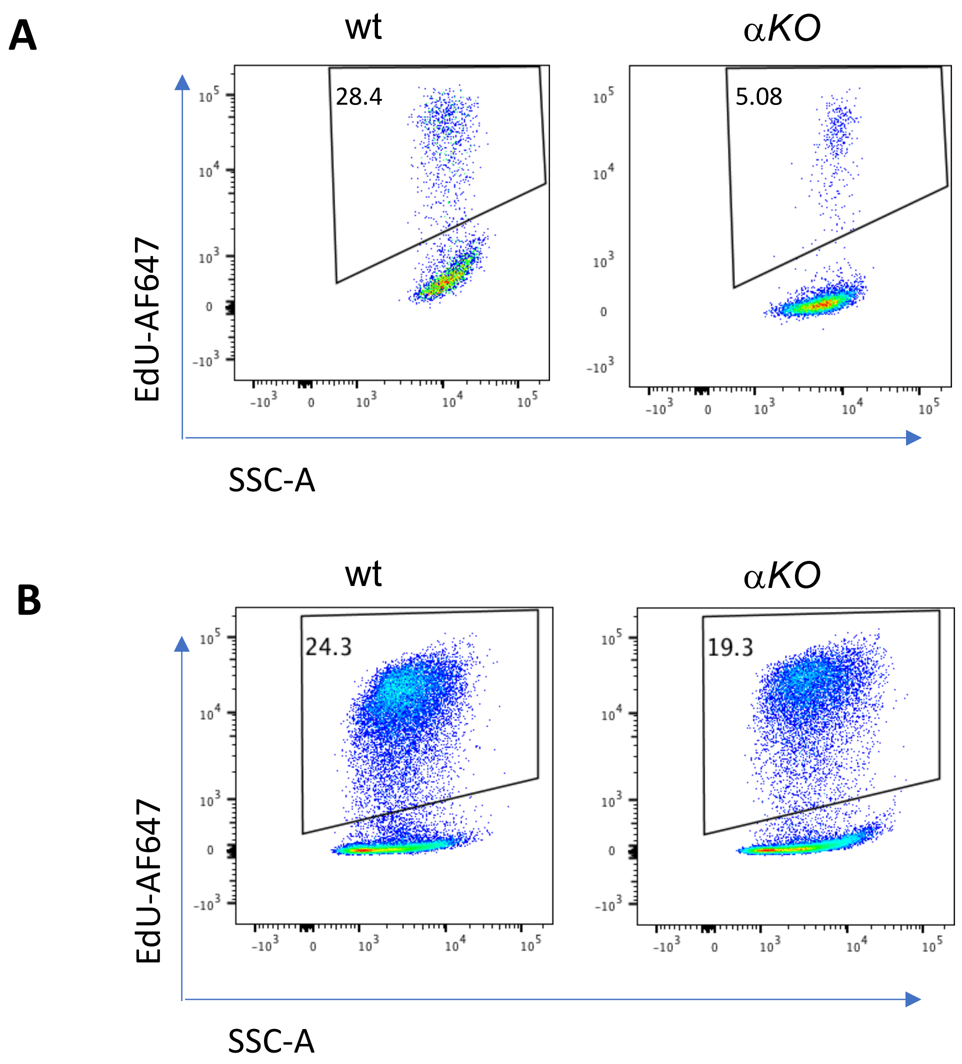

Supplemetary Figure 3. Flow cytometry analysis of EdU incorportation in NSC/NPCs *in vitro* and *in vivo*. Representative plots for cell subset of EdU+ cells *in vitro* (A, related to Figure 4C) and *in vivo* (B, related to Figure 4D).

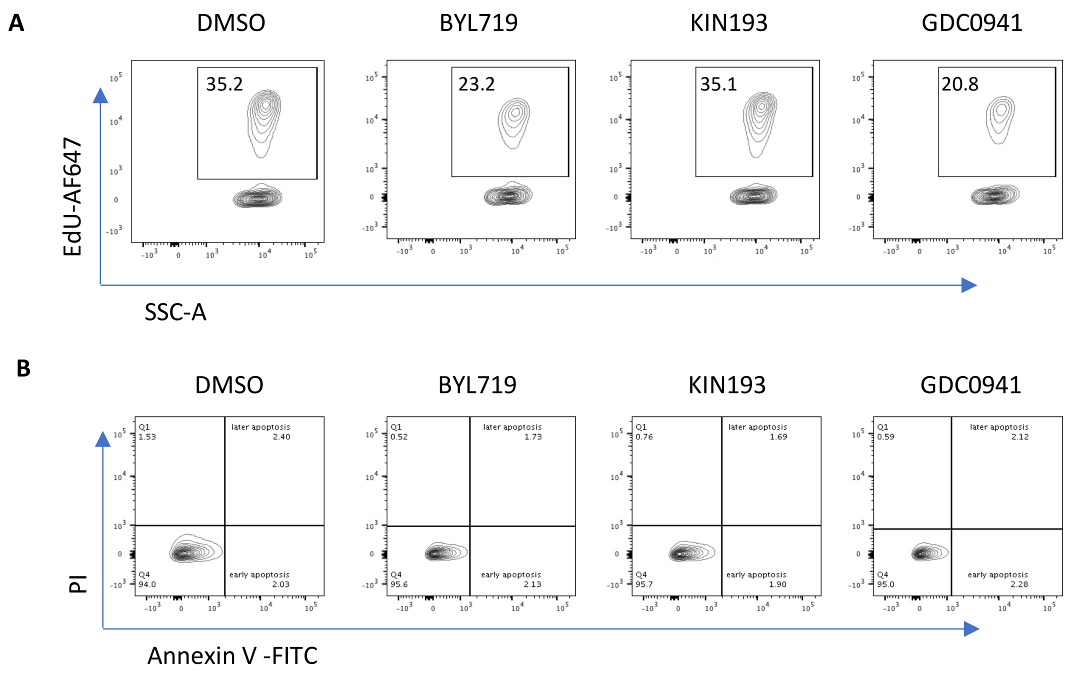

Supplemetary Figure 4. Flow cytometry analysis of EdU incorporation (A) and AnnexinV/PI apoptosis (B) in wild type NSC/NPCs with indicated PI3K inhibitors treatment. Representative plots of cell subset of NSC/NPCs related to Figure 4F and 4G.
